# A Worthy Foundation

**Published:** 1869-11

**Authors:** 


A Worthy Foundation.—The Consumptives' Home, at Boston, which was established on September 28th, 1864, is an institution supported upon unusual principles. There is no fund endowment or known pecuniary provision whatever existing for the support of the Home. No human friend has ever made any promise, express or implied, to sustain the institution, which is supported entirely by voluntary gifts obtained through *prayer*. The only means of soliciting money or aid in food, clothing, or any of the necessaries of life, have been the prayers of the founder and manager. The journal of this benevolent physician contains interesting accounts of the numerous occasions when the Home was reduced to the last extremity through the want of some particular article, and when that identical article was sent to the institution in answer to prayers for it. The Consumptives' Home was started with one house, but now occupies four buildings, the purchase money for which was raised in answer to prayer. The last report of the institution shows $50,328 has been given by friends during the last five years, and $20,105 during the past year.

The Home has since its opening accommodated 331 patients, and accommodated 165 patients during the year ending September, 1869.

